# cGAS-STING Pathway Does Not Promote Autoimmunity in Murine Models of SLE

**DOI:** 10.3389/fimmu.2021.605930

**Published:** 2021-03-29

**Authors:** Mona Motwani, Jason McGowan, Jennifer Antonovitch, Kevin MingJie Gao, Zhaozhao Jiang, Shruti Sharma, Gretchen A. Baltus, Kevin M. Nickerson, Ann Marshak-Rothstein, Katherine A. Fitzgerald

**Affiliations:** ^1^Program in Innate Immunity, Division of Infectious Diseases and Immunology, Department of Medicine, University of Massachusetts Medical School, Worcester, MA, United States; ^2^Merck & Co., Inc., Kenilworth, NJ, United States; ^3^Department of Immunology, University of Pittsburgh, Pittsburgh, PA, United States; ^4^Division of Rheumatology, Department of Medicine, University of Massachusetts Medical School, Worcester, MA, United States

**Keywords:** cGAS/STING, SLE, pristane, DNaseI, TLRs

## Abstract

Detection of DNA is an important determinant of host-defense but also a driver of autoinflammatory and autoimmune diseases. Failure to degrade self-DNA in DNAseII or III(TREX1)-deficient mice results in activation of the cGAS-STING pathway. Deficiency of cGAS or STING in these models ameliorates disease manifestations. However, the contribution of the cGAS-STING pathway, relative to endosomal TLRs, in systemic lupus erythematosus (SLE) is controversial. In fact, STING deficiency failed to rescue, and actually exacerbated, disease manifestations in Fas-deficient SLE-prone mice. We have now extended these observations to a chronic model of SLE induced by the i.p. injection of TMPD (pristane). We found that both cGAS- and STING-deficiency not only failed to rescue mice from TMPD-induced SLE, but resulted in increased autoantibody production and higher proteinuria levels compared to cGAS STING sufficient mice. Further, we generated cGAS^KO^Fas^lpr^ mice on a pure MRL/Fas^lpr^ background using Crispr/Cas9 and found slightly exacerbated, and not attenuated, disease. We hypothesized that the cGAS-STING pathway constrains TLR activation, and thereby limits autoimmune manifestations in these two models. Consistent with this premise, mice lacking cGAS and Unc93B1 or STING and Unc93B1 developed minimal systemic autoimmunity as compared to cGAS or STING single knock out animals. Nevertheless, TMPD-driven lupus in B6 mice was abrogated upon AAV-delivery of DNAse I, implicating a DNA trigger. Overall, this study demonstrated that the cGAS-STING pathway does not promote systemic autoimmunity in murine models of SLE. These data have important implications for cGAS-STING-directed therapies being developed for the treatment of systemic autoimmunity.

## Introduction

Systemic lupus erythematosus (SLE) is a heterogeneous autoimmune disease with a number of clinical manifestations including systemic inflammation, development of pathogenic antibodies, deposition of immune complexes and finally end organ damage (1). Genetic predisposition, environmental factors as well as both innate and adaptive arms of the immune system play an important role in initiation and amplification of this disease. Innate immune sensors present in endosomal compartments and in the cytosol, such as TLR9 and cGAS, respectively, can detect both microbial and host nucleic acids. It's been established in numerous murine models, that the endosomal TLRs, TLR7, and TLR9, play a critical role in SLE and related systemic autoimmune diseases (2, 3), while the role of STING in SLE is more controversial. Importantly, loss of function (LOF) mutations in the extracellular DNAse, DNAse1L3, originally identified in SLE patients (4–6), results in the accrual of DNA/RNA-associated microparticles in the blood (7–9). In mice, genetic deletion of DNAse1L3 promotes an SLE-like disease through a mechanism that is dependent on TLR7 and TLR9 and not STING (7, 9).

By contrast, LOF mutations of cytosolic DNAses such as DNAse II or DNAse III (Trex1) in patient populations are associated with systemic monogenic autoinflammatory diseases (10, 11). Genetic deletion of these cytosolic nucleases in mice, leads to embryonic lethality (DNAse II) (12) or myocarditis (TrexI) (13) through mechanisms dependent on the cGAS-STING pathway (14, 15). These data suggest that SLE is driven by extracellular DNA that is delivered to endosomal TLRs through receptors such as the BCR, LL37, or FcγRs while monogenic autoinflammatory diseases are driven by the aberrant accumulation of DNA in the cytosol that is detected by the cGAS/STING pathway. Autoantibody production has been detected in both Trex1 and DNAse II/IFNAR double deficient mice however, autoantibodies in DNAse II/IFNAR double defiicent mice have been shown to be Unc93B1 dependent (16). In addition, autoimmunity in DNAseIL3-deficient mice is dependent on endosomal MyD88 and endosomal TLRs (17).

Nevertheless, the cGAS/STING pathway has been postulated as a driver of lupus pathogenesis, although direct evidence for this is still limited. STING was recenlty implicated in the autoimmune phenotypes that develop in FcγR2-/- mice on a 129 background. However, since these mice were intercrossed with B6 STING^gt^ mice, the potential contribution of 129-associated risk alleles linked to the STING locus need to be considered (18). cGAMP, the cyclic dinucleotide generated by cGAS that activates STING, has been shown to be modestly elevated in the serum of a limited number of SLE patients (19). Most recently, UVB exposure which can drive lupus flares leads to an elevated type I IFN-I gene signature in the skin of mice which is dependent on the cGAS pathway and enhanced when cGAMP hydrolysis is blocked (20). Elevated STING activity has further been associated with increased expression of type I IFNs and ISGs (21, 22) and the degradation of damaged mitochondrial DNA by autophagy in patient populations (23). Moreover, while point mutations in human TREX1, that eliminate catalytic activity, cause the autoinflammatory disease Aicardi-Goutieres, point mutations in the N-terminus of TREX1, that retain DNAse activity, are associated with SLE and other unrelated conditions (24). The N-terminal mutations disrupt STING localization, and also disrupt the activity of the ER-resident enzyme oligosaccharyltransferase (OST) (25). In the absence of OST activity, immunogenic glycans accumulate in the ER and are the likely explanation for the association between the N-terminal mutations and SLE (24).

Murine models of C-terminal and N-terminal TREX1 mutations also develop distinct phenotypes. Patients heterozygous for a mutation that abrogates TREX1 catalytic activity, D18N, develop a skin disease that resembles familial chilblain lupus, while mice homozygous for this mutation develop myocarditis, multi-organ inflammation and autoantibody production, similar to TREX1^−/−^ mice (26). By contrast, mice homozygous for C-terminal frameshift mutation, D272fs, previously associated with SLE (27), exhibit no clinical manifestations of disease but do develop high autoantibody titers primarily directed against non-nuclear antigens, and thus distinct from the autoantibody specificities associated with SLE (28). Therefore, the association between TREX1 mutations and murine lupus is weak.

We previously generated STING/Fas double deficient autoimmune prone mice and found that STING deficient mice developed more severe, not less severe, disease compared to Fas-deficient autoimmune prone controls (29). Mechanistically, we showed that STING-deficient macrophages expressed decreased levels of negative regulators of immune activation and therefore were hyper-responsive to TLR ligands (29). Together, our data indicated that the STING pathway does not promote, but rather constrains, TLR-driven lupus-like diseases. However, our prior work used STING-deficient autoimmune mice that were generated by intercrossing STING-deficient B6/129 and MRL/Fas^lpr^ mice; a limitation to these F2 studies was the potential contribution of risk alleles derived from the B6/129 background, that could be linked to the STING locus in the STING-deficient mice. Moreover, a critical question that remained unexplored was whether the regulatory effects of STING were dependent on the upstream DNA sensor cGAS. To address these issues, we have now further defined the role of STING and cGAS by directly targeting MRL/lpr mice using CRISPR/Cas9 genome editing. There has also been some concerns in the literature regarding the relevance of the MRL/lpr model to human disease. To address this concern, we have also expanded our analysis to the chronic model of TMPD-induced SLE in both cGAS- and STING-deficient B6 mice. We found that neither cGAS- nor STING- deficiency protected MRL/lpr mice. We also found that neither cGAS- nor STING-deficiency prevented TMPD-induced lupus. These studies further confirm our original observations and demonstrate that STING does not promote murine SLE in either genetically programmed or inducible models of SLE.

One caveat to the TMPD model is that it is thought to be driven predominantly by the RNA-sensing TLR, TLR7 (30, 31) bringing into question the actual role of DNA sensors in these mice. To evaluate the potential role of DNA sensors in the TMPD model, we overexpressed DNAseI using an AAV9 gene therapy approach by injecting an AAV9-DNAse I vector i.p., prior to the TMPD inoculation. The DNAseI expressing mice developed much less severe clinical manifestations than mice injected with the vector control. Thus, DNA contributes to systemic inflammation in TMPD injected mice, along with TLR7 ligands. Overall, our study shows that the cGAS-STING pathway is not a driver of disease in TLR-dependent models of SLE, but instead constrains disease activity, presumably by limiting TLR activation. Nonetheless, DNA sensing still plays an important role in mediating autoimmune pathologies and depleting extracellular DNA can rescue TMPD-injected mice from SLE.

## Materials and Methods

### Mice

Wild-type C57BL/6 mice were purchased from the Jackson Laboratory. STING^KO^ mice, fully backcrossed to the C57BL/6 background, were kindly provided by Dr. Daniel Stetson University of Washington, Seattle, WA (32). cGAS^KO^ mice on a B6 background were generated using cryopreserved embryos obtained from the European Conditional Mouse Mutagenesis Program (EUCOMM). cGAS^KO^ MRL/MpJ-*Fas*^*lpr*^ mice were generated via CRISPR/Cas9 genome editing at Merck & Co., Inc., Kenilworth, NJ using two gRNA sequences “*AGGACCAGAACACCTTGTAG*” and “*TGACCGCACGACTTACCCTG*” targeting exon 2. The deletion of exon 2, resulting in 581bp deletion, was confirmed using common forward primer 5′CCTAGCCTTGGCTATGTGGT3′ and reverse primer 5′AACAGTTCTAAATAACCGCTTTCG3′ for WT and 5′GAGCTGTAGATGCCCAAGTG3′ reverse primer for cGAS^KO^. Unc93B1^KO^ mice were provided by Eicke Latz, University of Massachusetts Medical School, Worcester, MA (33). All mice were bred and maintained at the Department of Animal Medicine of the University of Massachusetts Medical School in accordance with the regulations of the American Association for the Accreditation of Laboratory Animal Care, and all protocols were approved by the Institutional Animal Care and Use Committee.

### TMPD and AAV Injection

12–16-week-old mice received a single intraperitoneal injection of 500 ul of 2,6,10,14-Tetramethylpentadecane (TMPD, Sigma) and were analyzed 5–6 months after TMPD injection (34). In some experiments, C57BL/6 mice were injected with either AAV9-GFP or AAV9-DNAse1 10 weeks prior to TMPD injections (35). Each mouse received 10^11^ virus particles in 200 uL PBS delivered by intraperitoneal injection and AAV expression was measured by qPCR.

### Flow Cytometry

Peritoneal exudate was collected in Hanks' Balanced Salt Solution (HBSS) media. Single cell suspensions obtained from the peritoneal cavity were stained with the following antibodies: anti-CD11b PercpCy5.5, anti-F4/80 PECy7, anti-Ly-6C APC, anti-Tim4 PE and anti-Ly-6G FITC. Flow cytometric analysis was carried out using a BD LSRII with Diva software (BD) and FlowJo Software (Tree Star). Live and single cells were gated to identify CD11b^+^ cells which were then gated to identify Ly6C^hi^ monocytes and Ly6G^hi^ granulocytes. Macrophages post TMPD injections were identified as live and single cells that were Ly6C^−^ Ly6G^−^ Tim4^−^ CD11b^+^ and F4/80^hi^ expressing cells. Splenocytes were stained with anti-CD11c pacific blue, SiglecH PE, anti-Bst2 APC, anti-CD3 PerCPCy5.5, anti-B220 Pacific blue, anti-CD4 PECy7 and anti-CD8 PE.

### Generation of Bone Marrow Derived Macrophages

Bone marrow extracts were differentiated *in vitro* into bone marrow derived macrophages (BMDMs) in the presence of L929 supernatants for 7 days. The rested BMDMs were then transfected with ISD (interferon stimulatory DNA) (5 μM) overnight using Lipofectamine 2000 from Invitrogen.

### Measurement of Serum Autoantibodies

HEp-2 human tissue culture substrate slides (MBL Code # ANK-120) were incubated with serum samples and bound antibodies were detected with DyLight 488-coupled detecting reagents as described in Christensen et al. (2). Anti–nucleosome concentrations were measured by ELISA with absorbance at 405/630 nm and compared with PL2-3 (in-house) as previously described (2). Autoantibodies reactive with dsDNA were measured by ELISA. Calf thymus DNA (Sigma) treated with S1 nuclease (ThermoFisher) was incubated for 1 h at room temperature on to poly-L-lysine treated ELISA plates. 1:50 diluted mouse sera was used and goat anti-mouse IgG H&L (HRP) (ab205719) was used as a secondary antibody. Anti-ds DNA antibody (ab27156) was used as standard.

### Proteinuria and Cytokine Measurement

An anti-mouse albumin ELISA kit was used to measure urine protein as per manufacturer's protocol (Bethyl Laboratories). Serum cytokine levels were determined using ELISA kits as per manufacturer's protocol: anti-mIFNγ and anti-mIL-17 (BD Biosciences), anti-mIL-10 (eBioscience) and anti-mTNFa (R&Dsystems). The IFN-β level in media supernatant was determined as previously described (36).

### Nanostring Analysis

Total RNA was isolated (Qiazol; Qiagen) and quantitated via a Nanodrop ND-1000 spectrophotometer (Thermo Scientific). Next, 50 ng of RNA was hybridized and quantified with a custom probe set using the NanoString nCounter analysis system (NanoString Technologies). Gene-expression data were normalized to housekeeping genes. All values were scaled by a log2(x – min(x) + 1) function and a heatmap generated using R-based software.

### Western Blot

Cells were lysed in 50 μL of ice-cold Pierce RIPA lysis buffer (ThermoFisher Scientific) supplemented with 1× complete protease and phosphatase mixture inhibitor (Sigma). Protein concentration was measured using a protein DC assay kit. Whole-cell lysates were denatured for 5 min at 85 °C in presence of 1× Sample Buffer and reducing agent (Invitrogen). Fifty micrograms of samples were separated by SDS/PAGE on 10% gels. Each gel was run initially for 15 min at 80 V and then at 120 V. Transfer onto nitrocellulose membranes (Bio-Rad) was done using a Trans-Blot Turbo Transfer system for 10 min. Membranes were blocked for 1 h with 5% skim-milk (Sigma Aldrich) at room temperature in PBS supplemented with 0.05% Tween-20 (PBST). Membranes were probed overnight at 4°C with the following primary antibodies: anti-cGAS (31659; CST) and anti–beta-actin Peroxidase (A3854; Sigma-Aldrich). All membranes were washed with PBST and exposed using the SuperSignal West Pico PLUS chemiluminescent substrate (ThermoScientific) on ImageQuant LAS4000 mini Imager (GE Healthcare).

### H & E Staining of Tissues

All tissues were fixed in 10% neutral buffered formalin for 24–48 h before being processed and paraffin-embedded. Five-micrometer-thin sections were stained by H&E in an automated stainer (Leica Autostainer XL). Histomorphology of each H&E slides was evaluated by Applied Pathology Systems at low and high-power field on Olympus BX40 microscope, and the images were captured with Olympus cellSens Entry software at indicated magnifications.

### Statistical Analysis

All data were analyzed by non-parametric Mann-Whitney test using GraphPad Prism Software (GraphPad Software, San Diego, CA). Experiments are reported as mean +/– SEM. Differences are designated as one asterisk if *p* < 0.05, as two asterisks if *p* < 0.01 and three asterisks if *p* < 0.001.

## Results

### Overexpression of DNAseI Enzyme Ameliorates Inflammation in Chronic TMPD Mediated Peritonitis

TMPD (2,6,10,14-Tetramethylpentadecane) or pristane is a naturally occurring hydrocarbon oil, which when introduced into the peritoneal cavity induces features of SLE in non-autoimmune prone mice (34). These include systemic inflammation, autoantibody production and glomerulonephritis. TMPD-driven lupus in B6 mice is highly dependent on TLR7 (30, 31), while the role of TLR9 is less straightforward. In general, TLR9 is required for the production of anti-dsDNA autoantibodies (2), but TLR9^−/−^ SLE-prone mice invariably develop more severe autoimmunity, and TLR9^−/−^ TMPD-injected BALB/c mice develop much more severe renal disease than their TLR9-sufficient littermates (37). Since TMPD induces cell death, DNA- and RNA-associated-cellular debris is likely to accumulate in the peritoneal cavity and other tissues throughout the body (34, 38). In addition, dysregulation of extracellular enzymes like DNAseI and DNAse1L3 that degrade DNA and DNA-associated complexes (microparticles) released from dying cells have also been implicated in SLE (9). DNAse1 is the most abundant secreted endonuclease, that is primarily expressed in the salivary glands, kidneys and gut. To explore the role of extracellular DNA in TMPD-induced lupus, we overexpressed DNase I using AAV9 that leads to widespread expression in order to test whether it could limit DNA uptake and thereby prevent or enhance clinical manifestations of SLE.

**Figure 1:**
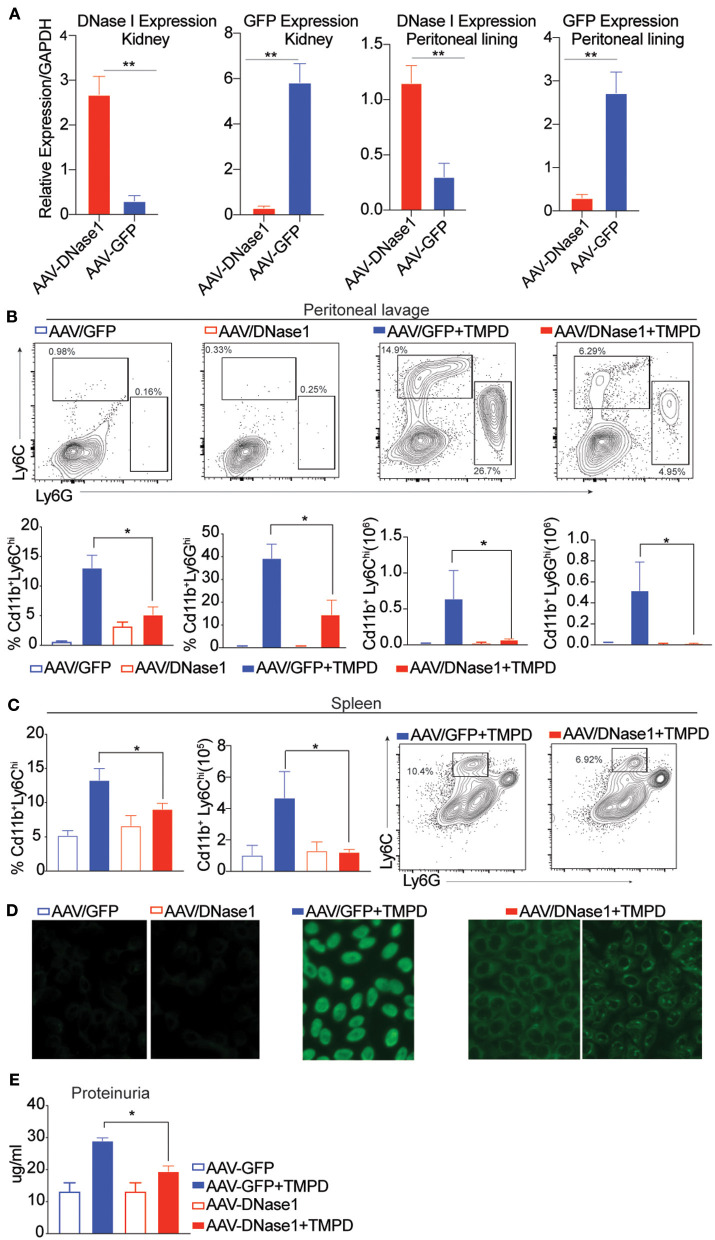
Overexpression of DNAse I ameliorates inflammation in chronic TMPD mediated peritonitis. 8–10 weeks old female C57BL6/J WT mice were either injected i.p. with 10^11^ AAV/GFP or AAV/DNAseI particles in 200 uL PBS. 10 weeks later mice were either injected with 500 ul TMPD or un-injected and analyzed from two independent experiments 6 months after TMPD injection. All the experiments were performed using the following N's of mice- AAV/GFP (blue open bars) AAV/DNAseI (red open bars) are uninjected mice *n* = 3. AAV/GFP+TMPD (blue closed bars) *n* = 4 and AAV/DNAseI +TMPD (red closed bars) are injected mice *n* = 8. **(A)** qPCR was performed to measure DNAse1 and GFP gene expression in total tissue, peritoneal lining and kidney. AAV/DNAseI injected mice are indicated in red bars and AAV/GFP mice are indicated in blue. **(B)** CD11b^+^ peritoneal exudate cells stained to identify Ly6C^hi^ monocytes and Ly6G^hi^ granulocytes. AAV/GFP (blue) and AAV/DNAse I (red) uninjected mice are indicated by open bars and TMPD injected mice are indicated as closed bars. The top panel shows representative flow plots and frequency/ numbers are graphed in the bottom panel. **(C)** CD11b^+^ splenic cells stained to identify Ly6C^hi^ monocytes. AAV/GFP (blue) and AAV/DNAseI (red) uninjected mice are indicated by open bars and TMPD injected mice are indicated as closed bars. The right panel shows representative flow plots and frequency/numbers are graphed in the left panel **(D)** Sera was collected 6 months after TMPD injection and ANA was measured using HEp2 substrate slides. Representative image from uninjected mice and AAV/GFP +TMPD injected mice is shown. Representative image is shown for two patterns observed in AAV DNAseI TMPD injected mice at 20X magnification. **(E)** Urine samples collected 6 months after TMPD injection were screened for proteinuria using an albumin ELISA assay. AAV/GFP (blue) and AAV/DNAseI (red) uninjected mice are indicated by open bars and TMPD injected mice are indicated as closed bars. Statistical significance is represented by *P < 0.05, **P < 0.01.

B6 mice were first inoculated with AAV-9 expressing either GFP or DNaseI, and then injected with TMPD 10 weeks later. 6 months post-TMPD injection, these mice were evaluated for both DNAseI expression and features of systemic autoimmunity. The levels of DNAseI were examined in multiple tissues and found to be expressed at high levels in both the kidney and peritoneal lining in mice that were injected with AAV-9 expressing DNaseI as compared to mice that received AAV-GFP. Similarly, high level of GFP expression was detected only in mice that received AAV-GFP and not in AAV-DNAseI injected mice (Figure 1A). TMPD leads to an inflammatory infiltrate in the peritoneal cavity that includes both CD11b^+^ Ly6C^hi^ monocytes and CD11b^+^ Ly6G^hi^ granulocytes. We found that the frequency and total number of both CD11b^+^ Ly6C^hi^ inflammatory monocytes and CD11b^+^ Ly6G^hi^ granulocytes in the peritoneal exudate was dramatically decreased in the AAV/DNAseI+TMPD mice compared to the AAV/GFP+TMPD mice (Figure 1B). We also evaluated the spleen of these mice and observed reduced frequency and number of CD11b^+^ Ly6C^hi^ inflammatory monocytes in AAVDNaseI treated mice that received TMPD (Figure 1C). We next evaluated the autoantibody profiles of TMPD treated mice and observed that the mice expressing AAV DNAseI showed very different ANA patterns when compared to the AAV GFP treated mice. Fifty percent of the AAV/GFP+TMPD sera gave a homogenous nuclear staining pattern, indicative of autoantibodies reactive with dsDNA, while the sera from the AAV/DNAseI+TMPD mice gave either a cytoplasmic and/or a speckled nuclear pattern (Figure 1D). Finally, the AAV/DNAseI+TMPD mice developed less severe renal disease as indicated by proteinuria levels (Figure 1E). These results highlight the importance of DNA sensing in TMPD mediated systemic autoimmunity.

### STING and cGAS Deficiency Exacerbate Disease in a Chronic Model of TMPD Induced Autoimmunity

**Figure 2:**
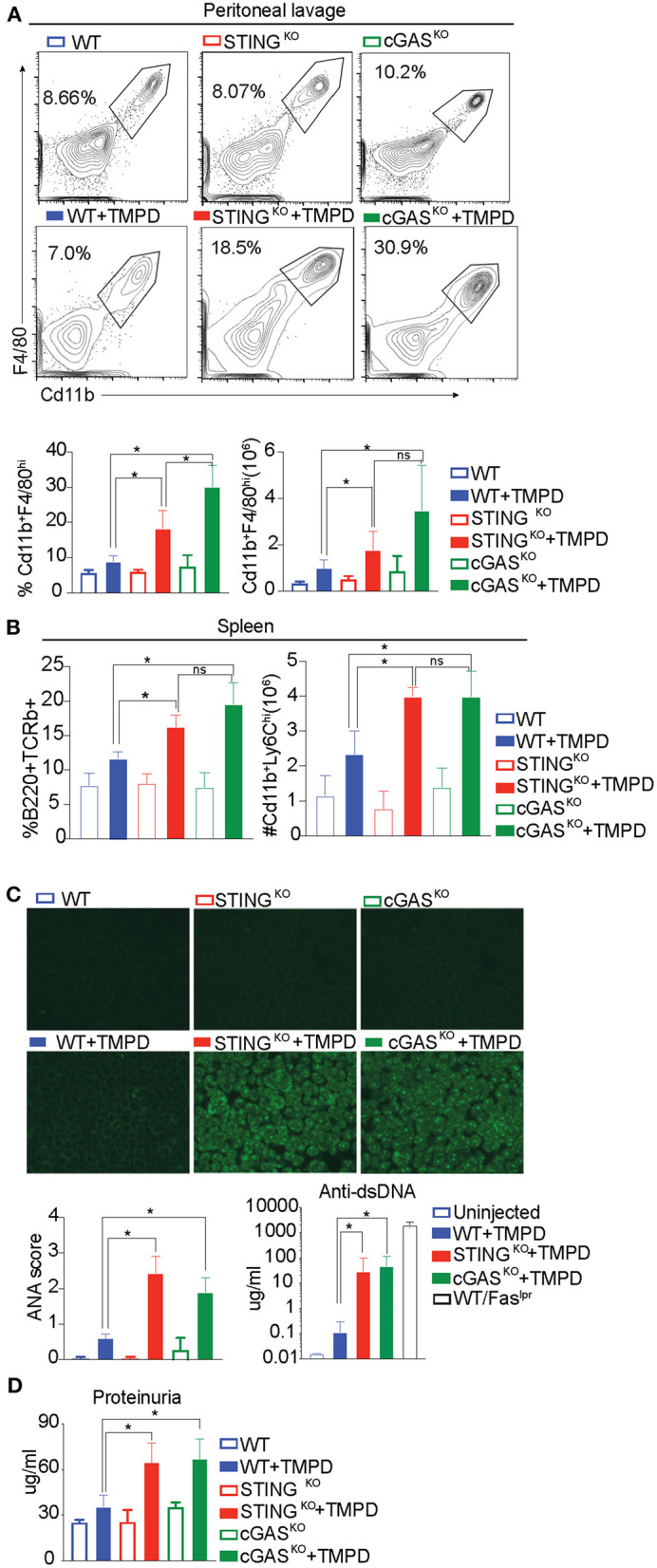
STING and cGAS deficiency exacerbate disease in chronic model of TMPD induced autoimmunity. 12–16 week-old female WT, STING^KO^ or cGAS^KO^ mice were either uninjected (open bars, *n* = 5) or injected with 500 ul of TMPD (closed bars, *n* = 15) were analyzed from two independent experiments. **(A)** 6 months later, peritoneal exudate cells were evaluated by flow cytometry. Live and single Ly6C^−^Ly6G^−^ cells were gated for CD11b^+^ and F4/80^hi^. The top panel indicates representative flow plots from uninjected mice (WT = blue open bar, STING^KO^ = green open bar and cGAS^KO^ = red open bar). The middle panel shows representative flow plots from injected animals (closed bars) and the graphs for both frequency and numbers of CD11b^+^ and F4/80^hi^ cells are shown in the bottom panel. **(B)** Flow cytometry was performed on splenocytes and double negative T cells (CD4^−^ CD8^−^) were identified as CD3^+^ B220^+^ and CD11b^+^ and Ly6C^hi^ cells were identified as inflammatory monocytes. The frequency of double negatives and cell numbers for inflammatory monocytes are graphed. **(C)** Sera was collected 3 months after TMPD injection and ANAs were detected using HEp2 substrate slides at 1:50 serum dilution. The representative images from each genotype and condition are shown at 20X magnification in the top panel. The slides were scored for fluorescence intensity and the scores are graphed at the bottom. Anti-ds DNA antibodies were measured in the serum at 3 months after TMPD injection (closed bars, *n* = 6). Sera from a WT/Fas^lpr^ strain was used as a positive control. **(D)** Proteinuria was assessed in urine collected 6 months after TMPD injection by using an albumin ELISA assay. Statistical significance is represented by *P < 0.05.

The development of autoimmunity progresses over a 6-month time course following administration of TMPD. To evaluate the role of STING and/or cGAS in disease progression, we injected WT, STING^KO^ and cGAS^KO^ with TMPD and assessed them 6 months later for various features of autoimmune disease. While B6 mice do not develop kidney pathology, they do develop immune cell abnormalities and modest proteinurea. We evaluated several parameters of inflammation, focusing first on the inflammatory infiltrate in the peritoneal cavity. We found a comparable frequency and number of CD11b^+^ Ly6C^hi^ monocytes and CD11b^+^ Ly6G^hi^ granulocytes in the TMPD-injected STING and cGAS deficient animals as in the TMPD-injected WT mice (Supplementary Figure S1A). However, within the cells that were CD11b^+^ Ly6C^−^ Ly6G^−^ we found a myeloid subset that was CD11b^+^ and F4/80^hi^ (Figure 2A). This subset of cells was Tim4^+^ in uninjected mice but Tim4^−^ in all TMPD injected mice indicating that these are not resident macrophages (Supplementary Figure S1B). This CD11b^+^F4/80^hi^ macrophage subset was increased in STING and cGAS deficient mice as compared to the WT mice both in frequency and number (Figure 2A). Several studies have reported distinct macrophage subsets to be pathological and major contributors of renal disease in both SLE prone patients and mice (39, 40). We also analyzed different immune cell subsets in the spleen and found that the numbers of Cd11b^+^ Ly6C^hi^ monocytes was increased in the spleen of STING and cGAS deficient pristane injected mice as compared to the WT. Moreover, we observed an increased percentage of CD3^+^ B220^+^ (CD8 CD4 double negative) T cells in the STING and cGAS deficient pristane injected mice as compared to the WT pristane injected mice (Figure 2B and Supplementary Figure S1C). These double negative cells are expanded in SLE patient populations and contribute to kidney disease (41, 42). Overall, we found increased frequency and number of cellular subsets that contribute to disease pathology both in the peritoneal lavage and in the spleen of the STING and cGAS deficient mice.

To further evaluate TMPD-injected WT, STING^KO^ and cGAS ^KO^ mice we examined serum for autoantibody production by immunofluorescent staining of HEp2 cells. Both STING- and cGAS-deficient mice developed detectable autoantibody levels by 12 weeks post TMPD injection, while the WT mice did not, as quantified in the bottom panel (Figure 2C left). We also quantified the production of anti-ds DNA antibodies in the serum and observed that the STING^KO^ and cGAS^KO^ TMPD injected animals had increased anti-dsDNA antibody titers as compared to WT injected mice (Figure 2C right). We also measured the status of serum cytokines in these animals and found increased levels of IL10, IL17, IFNγ and TNFα in STING^KO^ and cGAS^KO^ TMPD injected animals compared to WT mice (Supplementary Figure S1D).

Renal function was evaluated by assaying albumin titers in urine samples and both STING^KO^ and cGAS^KO^ injected mice showed evidence of increased proteinuria as compared to the WT injected mice (Figure 2D). Taken together, our data suggests that STING- or cGAS-deficient mice were not protected from TMPD-induced SLE, and even exhibited features of autoimmunity that were severe than their wild type counterparts.

### Exacerbation of TMPD-Induced SLE in cGAS or STING Deficient Mice Is Dependent on Endosomal TLRs

**Figure 3:**
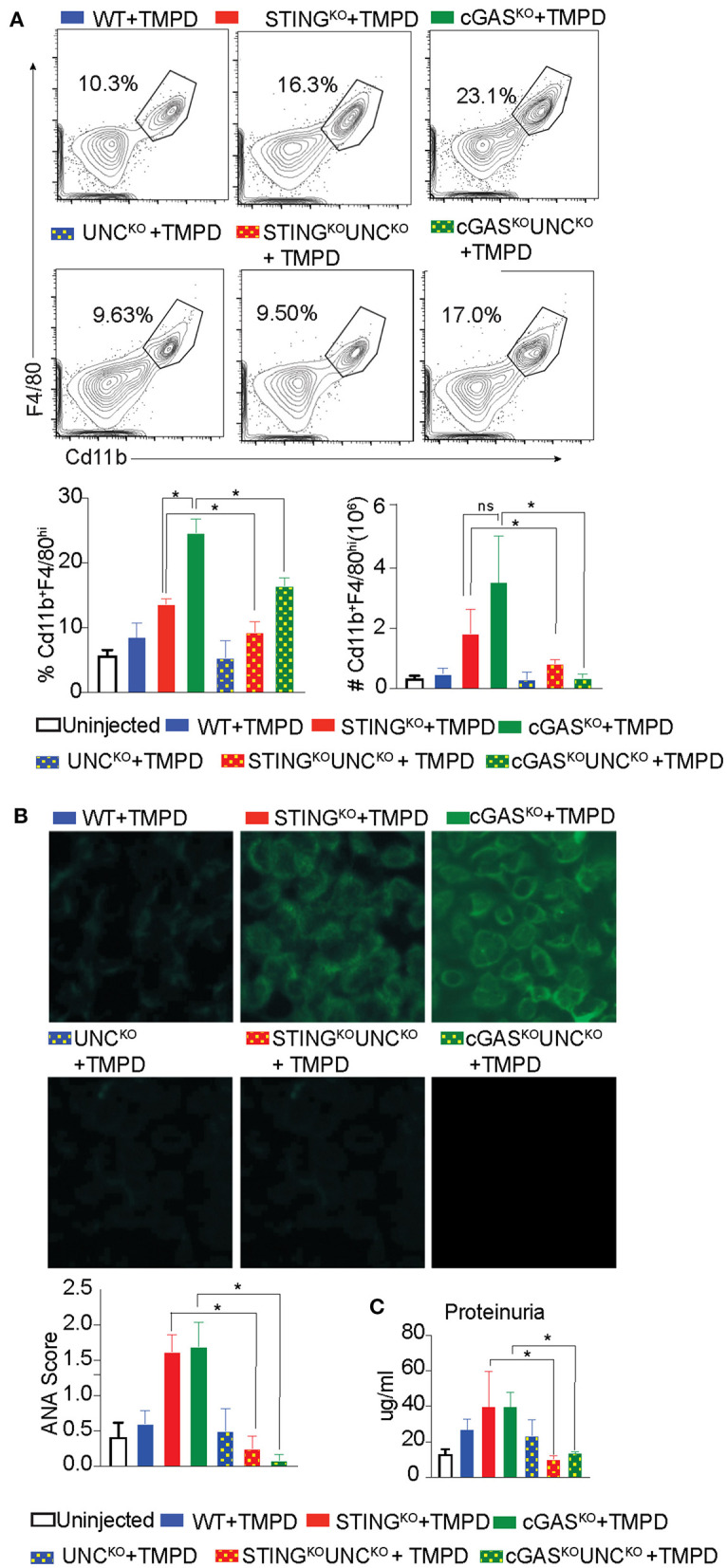
Exacerbation of TMPD-induced SLE in cGAS or STING deficient mice is dependent on endosomal TLRs. 12–16 week old female WT (blue), STING^KO^ (red), cGAS^KO^ (green), UNC^KO^ (blue with dots), UNC^KO^ STING^KO^ (red with dots), and UNC^KO^cGAS^KO^ (green with dots) mice were injected with 500 ul of TMPD (*n* = 10). Uninjected controls are indicated as open bars (*n* = 4 per genotype). The mice were analyzed 5 months post injection from two independent experiments. **(A)** Peritoneal exudate cells were evaluated by flow cytometry. Live and single Ly6C^−^Ly6G^−^ cells were gated for CD11b^+^ and F4/80^hi^. Representative flow plots for WT STING^KO^, cGAS^KO^ injected mice are shown in the top panel, the double deficient injected mice in the middle panel and the graphs for cell frequency and cell numbers are shown in bottom panel. **(B)** Sera was collected 3 months after TMPD injection and ANA were detected using HEp2 substrate slides at 1:50 serum dilution. The representative images from each genotype and condition are shown at 20X magnification in the top and middle panels and the scores for fluorescence intensity are graphed in the bottom. **(C)** Proteinuria was assessed in urine collected 5 months after TMPD injection by using an albumin ELISA assay. Statistical significance is represented by *P < 0.05.

Our previous study showed that STING deficient macrophages when stimulated with TLR9 and TLR7 ligands CpGB and CL097, respectively, produced increased levels of the proinflammatory cytokines IL6 and TNFα as compared to STING sufficient cells (29). This hyper-responsiveness correlated with reduced expression of genes involved in negative regulation of TLR signaling such as A20, suppressor of cytokine signaling 1 (SOCS1) and 3 (SOCS3) (29). To determine whether the STING-exacerbated features of SLE were dependent on endosomal TLRs *in vivo*, we generated STING^KO^ Unc93B1^KO^ and cGAS^KO^ Unc93B1^KO^ double deficient lines. We then injected single Unc93B1^KO^, the double deficient mice, and their Unc93B1-sufficient counterparts with TMPD, and evaluated them for features of systemic autoimmunity 5 months later. As above, the number of CD11b^+^ F/480^hi^ macrophages in the peritoneal cavity was increased in STING^KO^ and cGAS^KO^ mice as compared to WT mice and significantly reduced in cGAS^KO^ UNC93B1^KO^ and STING^KO^ Unc93B1^KO^ double-deficient mice (Figure 3A). We also found that again both STING^KO^ and cGAS^KO^ mice developed higher levels of autoantibody than the WT mice, but failed to produce autoantibodies if they were Unc93B1-deficient (Figure 3B). Moreover, the proteinuria levels of STING and cGAS deficient mice were significantly higher than the WT mice, but STING/Unc93B1 and cGAS/Unc93B1double deficient mice failed to develop detectable proteinuria (Figure 3C). Collectively, these findings indicate that the elevated features of autoimmunity observed in cGAS or STING-deficient mice was abrogated when endosomal TLR signaling was abolished by deletion of Unc93B1 *in vivo*.

### cGAS Deficiency Does Not Rescue SLE-Prone MRL/Fas^lpr^ Mice

**Figure 4:**
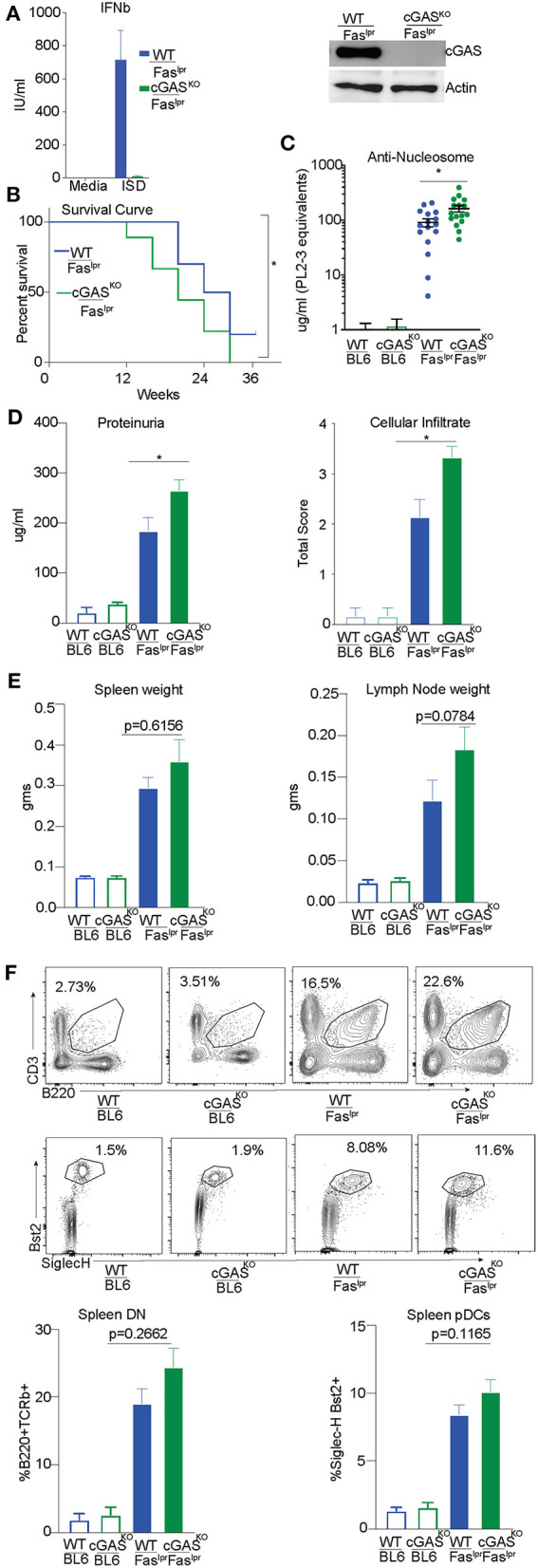
cGAS deficiency does not rescue SLE-prone MRL/Fas^lpr^ mice. **(A)** BMDMs were isolated from WT/ Fas^lpr^ and cGAS ^KO^/ Fas^lpr^ mice. These cells were either transfected or un-transfected with ISD 5 uM and interferon β ELISA was performed after overnight treatments. **(B)** BMDM cell lysates were prepared and western blot was performed to detect cGAS levels. Actin was blotted as loading control. **(B)** WT/Fas^lpr^ (*n* = 10) and cGAS^KO^/Fas^lpr^ (*n* = 18) mice were observed until the time of death and Kaplan Meier survival analysis was performed. **(C)** Anti-nucleosome ELISA was performed for 12–16 week old mice B6 WT and B6 cGAS^KO^ mice (open bars), WT/Fas^lpr^ littermates (blue bar, *n* = 16), cGAS^KO^/Fas^lpr^ mice (green bar, *n* = 16). **(D)** Urine samples from 16 week old B6 WT and B6 cGAS^KO^ mice (open bars), WT/Fas^lpr^ littermates (blue bar, *n* = 16), cGAS^KO^/Fas^lpr^ mice (green bar, *n* = 18) were screened for proteinuria using an albumin ELISA assay. H&E staining was performed to analyze cellular infiltrate WT/Fas^lpr^ littermates (blue bar, *n* = 7), cGAS^KO^/Fas^lpr^ mice (green bar, *n* = 7). **(E)** Spleen and lymph nodes weights were determined at 12 weeks of age (*n* = 16 mice each for WT/Fas^lpr^ and cGAS^KO^/Fas^lpr^). **(F)** Flow cytometry was performed on splenocytes and double negative T cells (CD4^−^ CD8^−^) were identified as CD3^+^ B220^+^. To enumerate pDCs, B220^−^ CD3^−^ CD11c^+^ splenocytes were gated for SiglecH^+^ and Bst2^+^ cells. Representative flow plot is shown for each cell type and corresponding genotype in the top/middle panels and the frequency of cells is graphed in the bottom. All the flow analysis was performed on 12 week old female mice, (*n* = 16 mice each for WT/Fas^lpr^ and cGAS^KO^/Fas^lpr^). Statistical significance is represented by *P < 0.05.

We had previously reported exacerbation of autoimmune features in STING-deficient Fas^lpr^ mice generated by intercrossing B6/129 STING-deficient mice with MRL/Fas^lpr^ mice (29). To avoid any risk alleles that could be linked to the cGAS locus and modify disease outcome, we directly generated cGAS deficient MRL/Fas^lpr^ mice using a CRISPR/Cas9 genome editing strategy in MRL/lpr mice. We confirmed cGAS deficiency in the gene-edited strain by stimulating cGAS-deficient and control bone marrow derived macrophages (BMDMs) with ISD and measuring interferon production. cGAS^KO/^ Fas ^lpr^ cells failed to produce Interferonβ (IFNβ) compared to WT/Fas^lpr^ cells in response to ISD, confirming that the CRISPR generated strain was functionally cGAS deficient (Figure 4A left). We also immunoblotted lysates obtained from WT/Fas^lpr^ and cGAS^KO^/Fas ^lpr^ BMDMs. As expected, we found no detectable levels of cGAS protein in macrophages from cGAS^KO^/Fas ^lpr^ mice whereas WT/Fas^lpr^ macrophages expressed cGAS protein (Figure 4A right). cGAS^KO^/Fas^lpr^ and WT/Fas^lpr^ littermate controls were then evaluated for various SLE features and WT and cGAS deficient mice on a C57BL/6 background were used as negative controls since they do not develop autoimmunity spontaneously. Survival of the cGAS^KO^/Fas^lpr^ mice was slightly compromised as compared to their WT/Fas^lpr^ littermates (Figure 4B). The cGAS^KO^/Fas^lpr^ mice developed significantly higher levels anti-nucleosome antibodies in the serum as compared to WT/Fas^lpr^ littermate controls (Figure 4C). The cGAS^KO^/Fas^lpr^ mice show increased proteinuria in the urine and increased cellular infiltrate in the kidneys as compared to the WT/Fas^lpr^ littermate controls (Figure 4D). However, splenomegaly or lymphadenopathy showed only trending increases in cGAS^KO^/Fas^lpr^ as compared to littermate controls (Figure 4E). Similarly, the percentage of CD3^+^ B220^+^ (CD8 CD4 double negative) T cells or pDC were only modestly increased in the cGAS^KO^/Fas^lpr^ mice compared to WT/Fas^lpr^ (Figure 4F). In addition, we also performed gene expression analysis in the total splenocytes obtained from these mice and we did not see any significant diffrences between the cGAS^KO^/Fas^lpr^ mice compared to WT/Fas^lpr^ (Supplementary Figure S2A). Importantly, our data indicates that neither STING nor cGAS deficiency rescue MRL/lpr mice from SLE and are therefore unlikely to sense the extracellular DNA that accumulates in this model. Thus, the importance of the cGAS/STING pathway in the promotion of autoinflammation does not necessarily translate to the promotion of SLE.

## Discussion

Engagement of cGAS and the endosomal DNA sensor TLR9, can lead to the production of type I IFNs and proinflammatory cytokines. Stressed, damaged, or dying cells can provide sources of endogenous ligands that can activate both pathways. The importance of TLRs in SLE disease pathogenesis has been demonstrated by both loss-of-function and gain-of-function mutations in multiple animal models of SLE (3, 43–45), whereas DNA sensing mediated by the cGAS-STING pathway has been implicated in several human monogenic autoinflammatory diseases (46–48), as well as in mouse models of type I interferonopathies (49–51). In contrast to autoinflammatory diseases, where STING activation amplifies inflammation, our initial study showed that the STING pathway can attenuate clinical manifestations of SLE (29). We have now extended our initial observation to a chronic inducible model of SLE (TMPD induced SLE) as well as gene-targeted MRL/lpr SLE-prone mice, where we can monitor the progression of the disease without the caveat of additional risk alleles due to mixed genetic backgrounds. Our findings show that STING or cGAS deficiency does not cure lupus in either case. Rather, consistent with our earlier work (29), STING or cGAS deficiency leads to an earlier onset of SLE as measured by autoantibody levels in the serum and proteinuria in the urine. These data provide a cautionary note for the use of cGAS-STING targeted therapies for the treatment of SLE.

We have now also shown that neither Unc93B1/STING nor Unc93B1/cGAS mice develop TMPD induced SLE. These data further demonstrate that even in mice that fail to express STING, SLE is still dependent on endosomal TLRs. To determine whether DNA plays a role as an instigator of TMPD-induced lupus, we generated mice with increased levels of extracellular DNAse I using a one-time i.p. injection of AAV encoding DNAse I. We initially set out to test the premise that the protective role of STING (and TLR9) was DNA-dependent, and if this were the case, depletion of DNA should have resulted in an amplified TLR7 signal and led to a more severe clinical outcome. Instead, DNAseI-AAV-treated mice did not make autoantibodies against dsDNA, as indicated by the ANA staining pattern, and did not even develop other clinical manifestations of SLE. These data point to a critical role for a DNA sensor, possibly TLR9, in promoting the development of TMPD-induced SLE, and suggest that there may be a bimodal interplay between TLR7 and TLR9 in disease initiation and then disease progression. Alternatively, a DNA sensor other than cGAS or TLR9 may be involved. DNAseI-treated mice injected with TMPD show an overall reduction in inflammation and proteinuria but a very different autoantibody profile which could be more RNA driven, a phenomenon similar to the impact of TLR9-deficiency. TLR9- and TLR7-deficient SLE prone mice fail to make autoantibodies reactive with DNA and RNA, respectively, while TLR7-deficient and TLR7/TLR9 double deficient mice develop much less severe clinical phenotypes and exhibit markedly prolonged survival (2). It is also important to mention, that although DNAse1 is the most abundant secreted endonuclease expressed in some tissues including the kidneys, the AAV gene delivery approach here could have led to a more widespread expression of DNAseI than that seen under physiological conditions.

Although the mechanisms remain unresolved, this study reinforces the importance of investigating both proinflammatory and anti-inflammatory activities of nucleic acid-sensors. Both the TLR9 and STING pathways promote the IFN- and NFκB-driven expression of proinflammatory cytokines and chemokines, but at the same time induce negative regulators of these pathways. Thus, the outcome of nucleic acid sensor engagement will depend on the balance between positive and negative regulators of inflammation and transcriptional regulation of the relevant genes which is likely to reflect activation status of the receptor expressing cells. The dual functionality for cytosolic pathways is not surprising since a published study showed that the cytosolic RNA sensor, RIG-I also dampens TLR responses in myeloid cells by suppressing TLR driven interleukin 12 (IL-12b) gene transcription (52). The evidence of increased autoantibody production due to STING deficiency is also seen in a DNAse1L3-deficient mouse model used to evaluate SLE, where DNAse1L3 another extracellular DNAse clears microvesicle-associated DNA. The DNAse1L3 knockout mice were crossed with STING-deficient mice and serum titers of anti-dsDNA IgG as determined by ELISA, were higher in DNAse1L3/ STING double knockout mice than in single DNAse1L3 knockout mice (7).

More recently, several groups have described a role for the STING pathway in the clearance of DNA by autophagy (53, 54), suggesting that STING deficiency could lead to reduced clearance of DNA. The ensuing accumulation of DNA in endosomal compartments of these cells could then enhance TLR dependent cytokine production through a cell intrinsic mechanism. DNAse II-deficient mice provide another example of excessive DNA accrual leading to the activation of multiple nucleic acid sensing pathways since the cGAS-STING, AIM2, and endosomal TLRs all have been shown to contribute to the development of autoimmunity or autoinflammation in this model (16, 55, 56). It is also possible that different DNA sensors may function in tissue specific manner for instance cGAS STING pathway can still contribute to certain aspects of disease such as the cutaneous lupus features as suggested by Skopelja-Gardner et al. (20). A better understanding of the interplay between cells responding to endogenous nucleic acid ligands will provide important insights for preventing the onset and progression of both autoinflammatory and autoimmune diseases in patients suffering from these conditions.

## Data Availability Statement

The original contributions generated for this study are included in the article/Supplementary Material and submitted to NCBI GEO (Gene Expression Omnibus) with accession number GSE169655 and linked to https://www.ncbi.nlm.nih.gov/geo/query/acc.cgi?acc=GSE169655. Further inquiries can be directed to the corresponding author/s.

## Ethics Statement

All mice were bred and maintained at the Department of Animal Medicine of the University of Massachusetts Medical School in accordance with the regulations of the American Association for the Accreditation of Laboratory Animal Care and all protocols were approved by the Institutional Animal Care and Use Committee.

## Author Contributions

SS, AM-R, and KAF designed research. SS, MM, JM, JA, KMG, and ZJ performed research. GAB and KMN contributed new reagents and analytic tools. MM, AM-R, and KAF wrote the paper. All authors contributed to the article and approved the submitted version.

## Conflict of Interest

GAB is employed by the company Merck and Co., Inc., Kenilworth, NJ, United States. The authors declare that this study received funding from sponsored research agreement from GSK (100251747). The funder had the following involvement with the study: decision to submit it for publication. The remaining authors declare that the research was conducted in the absence of any commercial or financial relationships that could be construed as a potential conflict of interest.
