# Supplementary figures and images for: cGAS-STING Pathway Does Not Promote Autoimmunity in Murine Models of SLE
Source: Front Immunol. 2021 Mar 29;12:605930. doi: 10.3389/fimmu.2021.605930 (PMC8040952; doi:10.3389/fimmu.2021.605930)

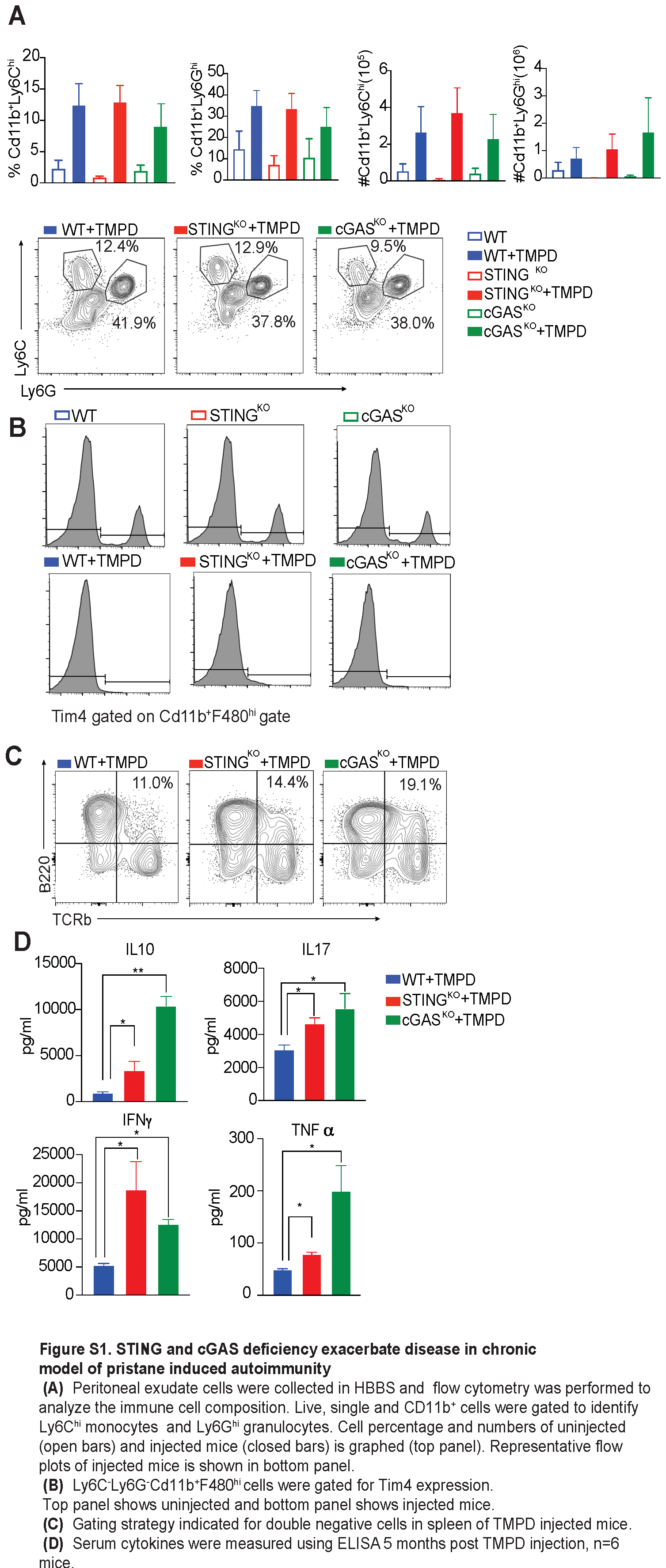

Supplement: Supplementary file 1 [file Image_1.tiff]

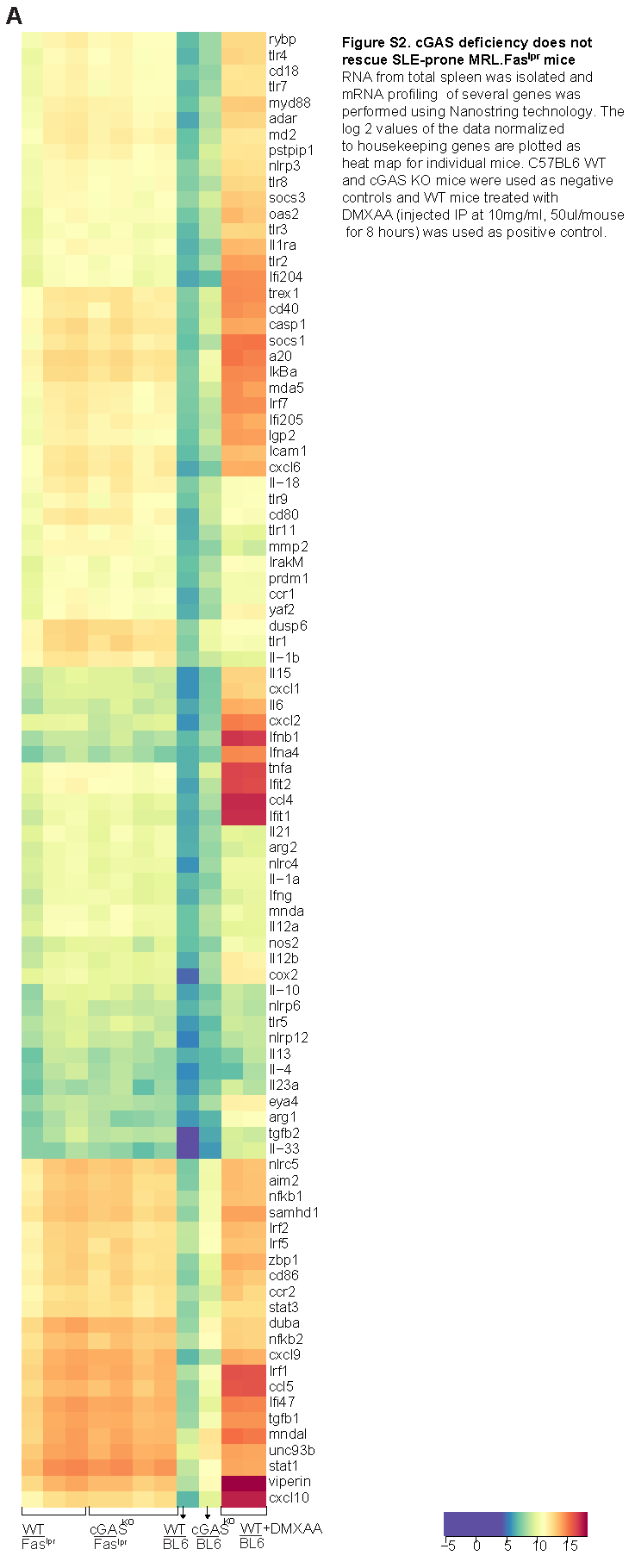

Supplement: Supplementary file 2 [file Image_2.tiff]
